# Rediscovering the Potential of Multifaceted Orphan Legume Grasspea- a Sustainable Resource With High Nutritional Values

**DOI:** 10.3389/fnut.2021.826208

**Published:** 2022-02-23

**Authors:** K. R. Ramya, Kuldeep Tripathi, Anjula Pandey, Surendra Barpete, Padmavati G. Gore, Archana Peshin Raina, Khalid Mahmood Khawar, Nigamananda Swain, Ashutosh Sarker

**Affiliations:** ^1^Division of Plant Genetic Resources, Indian Council of Agricultural Research-Indian Agricultural Research Institute, New Delhi, India; ^2^Indian Council of Agricultural Research-National Bureau of Plant Genetic Resources, New Delhi, India; ^3^International Center for Agricultural Research in the Dry Areas-Food Legume Research Platform, Amlaha, India; ^4^Department of Field Crops, Faculty of Agriculture, Ankara University, Ankara, Turkey

**Keywords:** climate-resilient, grasspea, multifaceted, orphan legume, sustainable

## Abstract

The genus *Lathyrus* consists of more than 184 herbaceous annual and perennial species suitable for multifaceted sustainable food and feed production system in the arid and semi-arid regions of the world. The grasspea is a promising source of protein nutrition. However, its potential is not being utilized fully due to the presence of neurotoxin content (β-N-oxalyl-l-α, β diaminopropionic acid, β-ODAP), a causal agent of non-reversible lower limbs paralysis. The high protein contents in seeds and leaves with ~90% digestibility make it sustainable super food to beat protein malnutrition in future. Therefore, it is desired to breed new grasspea cultivars with low β-ODAP contents. Limited research has been carried out to date about this feature. A draft genome sequence of grasspea has been recently published that is expected to play a vital role in breeding and identifying the genes responsible for biosynthesis pathway of β-ODAP contents in grasspea. Efforts to increase awareness about the importance of genus *Lathyrus* and detoxify β-ODAP in grasspea are desired and are in progress. Presently, in South Asia, systematic and dedicated efforts to support the farmers in the grasspea growing regions by disseminating low β-ODAP varieties has resulted in a considerable improvement in reducing the incidence of neurolathyrism. It is expected that the situation will improve further by mainstreaming grasspea cultivation by implementing different approaches such as the development and use of low β-ODAP varieties, strengthening government policies and improved detox methods. The present review provides insight into the multifaceted characteristics of sustainable nutritious grasspea in the global and Indian perspective.

## Introduction

The human population growth rate is very fast compared to the food yield per hectare as per the report of World Food Programme, 2018 (1). Therefore, there is a need to boost our food grain production by 70% (taking 2015 as the base year) to feed the 1.66 billion people and meet the Sustainable Development Goal (SDG) targets by 2030. Despite the fact that the hunger has decreased globally since 2000, the yield plateau in all major crops and increased malnutrition makes hidden hunger severe in many parts of world (2).The number of undernourished people in the world has continued to increase. If recent trends are not reversed, the SDG 2.1 zero hunger target will not be met. Sustainable development is only possible in communities where malnutrition is eradicated. The world may not achieve the global nutrition targets of ensuring access to safe, nutritious and sufficient food for all and eradicating all forms of malnutrition (3). Globally in 2020, the scale challenges in nutritional imbalances amounting to two billion people lacking key micronutrients like iron and vitamin A; 149 million children under age five were estimated to be stunted; 1.9 billion adults are overweight or obese, while 462 million are underweight (4) and out of 141 countries analyzed, 88% of countries face serious burden of more than one form of malnutrition and 29% have high levels all forms of malnutrition (stunted growth, obese and overweight) (5). Utilizing plant genetic resources of various climate-smart species, including underutilized and neglected crops, will be of great significance to achieve SDG's. The present communication reviews grasspea (*Lathyrus sativus* L.) a member of family Fabaceae (*Leguminosae*), subfamily *Papilionoideae*, and tribe *Vicieae*, which is an underutilized and neglected food, feed and pharmaceutically important crop that shows resistance to harsh environmental conditions (6, 7) like drought, heat, soil infertility, floods and many ranges of biotic stresses. It grows either as cultivated crop or weed under natural conditions in South, Southeast Asia, Middle East, Eastern Europe and in many other countries of the world. The cultivation of grasspea requires minimal inputs and cost; thereby, it can be successfully incorporated in the conservation agriculture and breeding programmes for developing climate smart (biotic and abiotic stress-resistant, nutrition rich) varieties. This crop deserves a sustainable and nutritionally rich status and therefore the rediscovery of its potential as food and nutritional security in reference to the global and Indian perspective is desirable.

## Origin and Domestication of Grasspea

**Figure 1:**
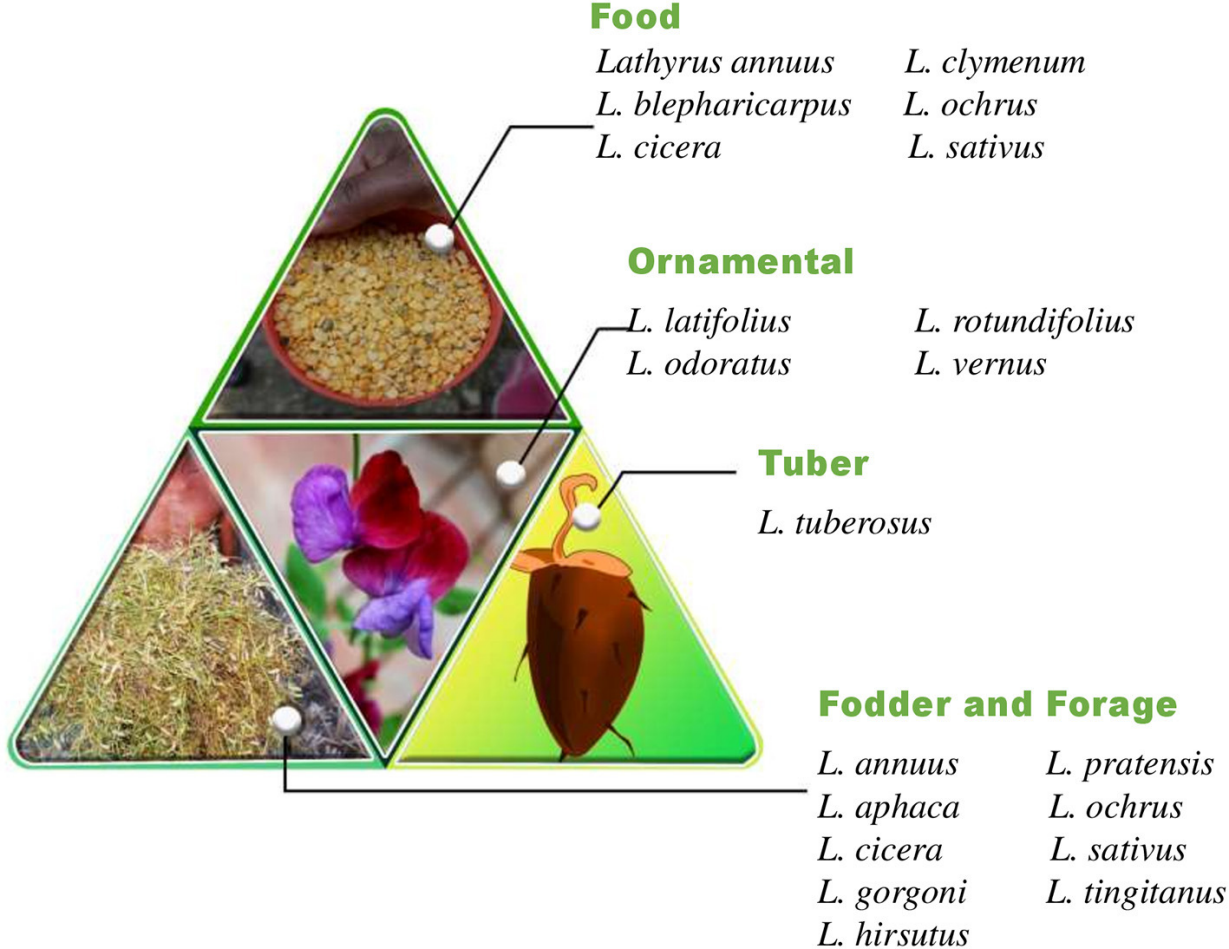
*Lathyrus* species and its uses (14, 15).

**Figure 2:**
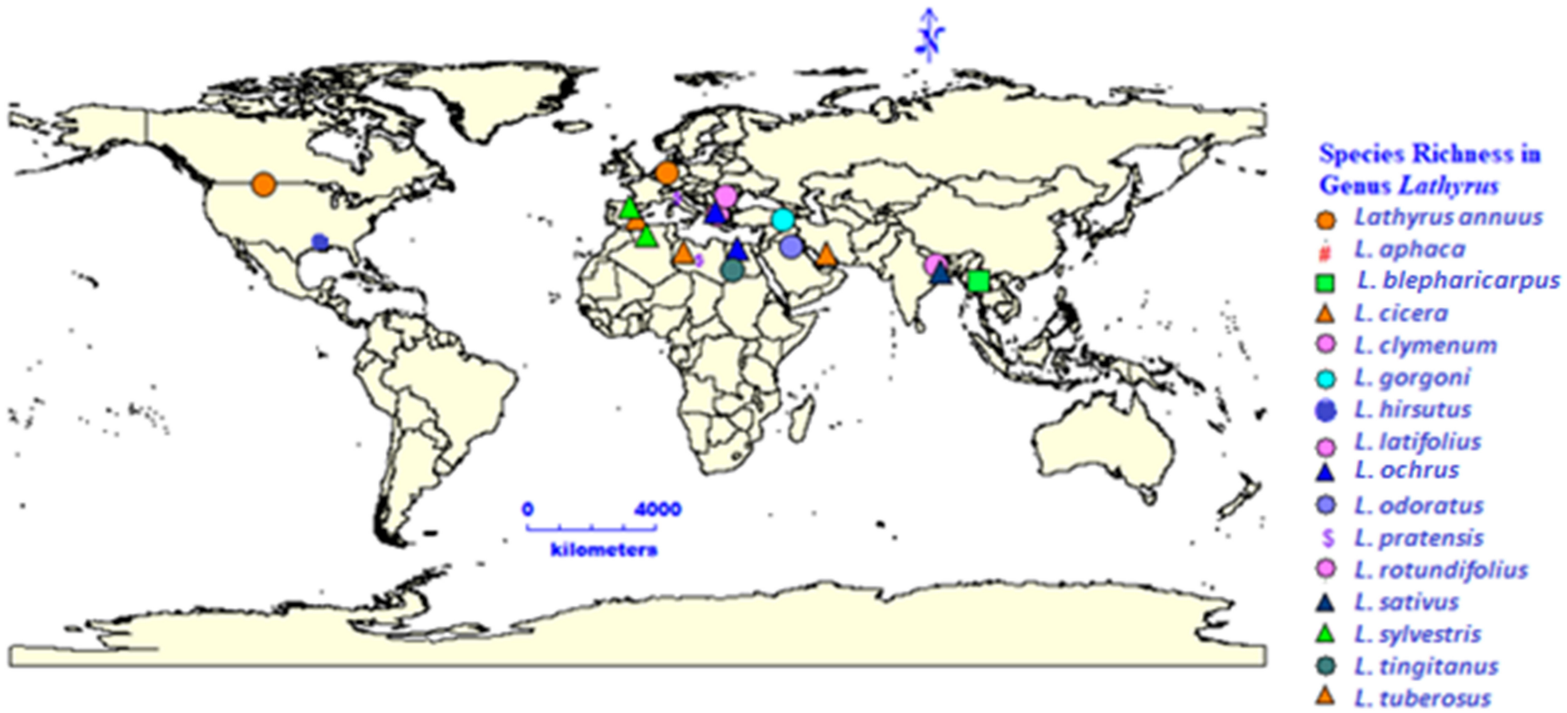
Species richness in genus *Lathyrus* and its distribution.

The word “*Lathyrus*” is derived from the ancient Greek word lathuros which means “exciting,” and refers to the aphrodisiac properties of grasspea (8). The grasspea is also known by many names (countries in parenthesis) like *chickling vetch, chickling pea, dog toothed pea* (America, Britain); *khesari* (Bangladesh); *san lee do* (China); *fovetta* (Cyprus); *sabberi* (Ethiopia); *gisette* (France); *khesari dal, lang, chural, latri, lakhori, batura, tiwra* (India); *pisellobrettone* (Italy); *kheshari* (Nepal); *matri, mattra, kesari* (Pakistan); *almorta* (Spain); *gilban* (Sudan); *murdumuk* (Turkey) and *pharetta, garbanzo* (Venezuela) (9). The current list of 184 taxonomically accepted names of the genus *Lathyrus* can be accessed through Plants of the World Online, Royal Botanic Gardens, Kew, United Kingdom. The genus *Lathyrus* consists of more than 160 annual and perennial species (10, 11) and subspecies (12) belonging to 15 divisions based on morphological features (13). The common uses of important species in the genus *Lathyrus* is given in Figure 1 and the global distribution of species in genus *Lathyrus* in the Figure 2 (14). *L. sativus, L. cicera* L., and *L. hirsutus* L. are the most extensively cultivated species for food and feed whereas *L. latifolius* L. and *L. odoratus* L. are gorgeous looking ornamental plants produced commercially in Europe (14, 15).

The cultivation and domestication of many important annuals such as wheat, pea, grasspea and lentil started during early neolithic era around the 6^th^ millennium Before Common Era (BCE) in the West Asia (Irano-Turanian regions). Later, these crops spread to the temperate Mediterranean region; moving further to tropics and sub-tropics in the northern hemisphere including East Africa, South Asia and naturalized in South America over time (16). Grasspea is grown as a pulse and fodder crop in Southeast Asian countries since time immemorial. The origin and distribution of crops have a long history and in several cases uncertainty is due to the discrepancies in the description of primary and secondary centers of diversity and natural spread in later stages (17). The earliest archaeological evidences dates back from the 8th millennium BCE to the Neolithic age from the village of Jamro lying at the foothills of the Zagros Mountains in Northern Iraq (close to the Turkish and Iranian borders) at an altitude of 800 msl (18, 19). The important archaeological evidences of grasspea are noted in adjacent areas of Tepe Sadz (7500–5700 BCE) and Ali Kosh (9500–7600 BC) in Iran (19) and in the Gangetic plains, India (2000–1500 BCE) (20) with presumption of its introduction from West Asia. Probable remains of *L. cicera* have also been reported at Azmaska Moghila, in Bulgaria 7000 BCE (21). Several archaeobotanical and phytogeographical evidences proved that grasspea was initially domesticated in the Balkan Peninsula during the early neolithic era, around the 6^th^ millennium BCE (22).

## Botany and Taxonomy of Grasspea

**Figure 3:**
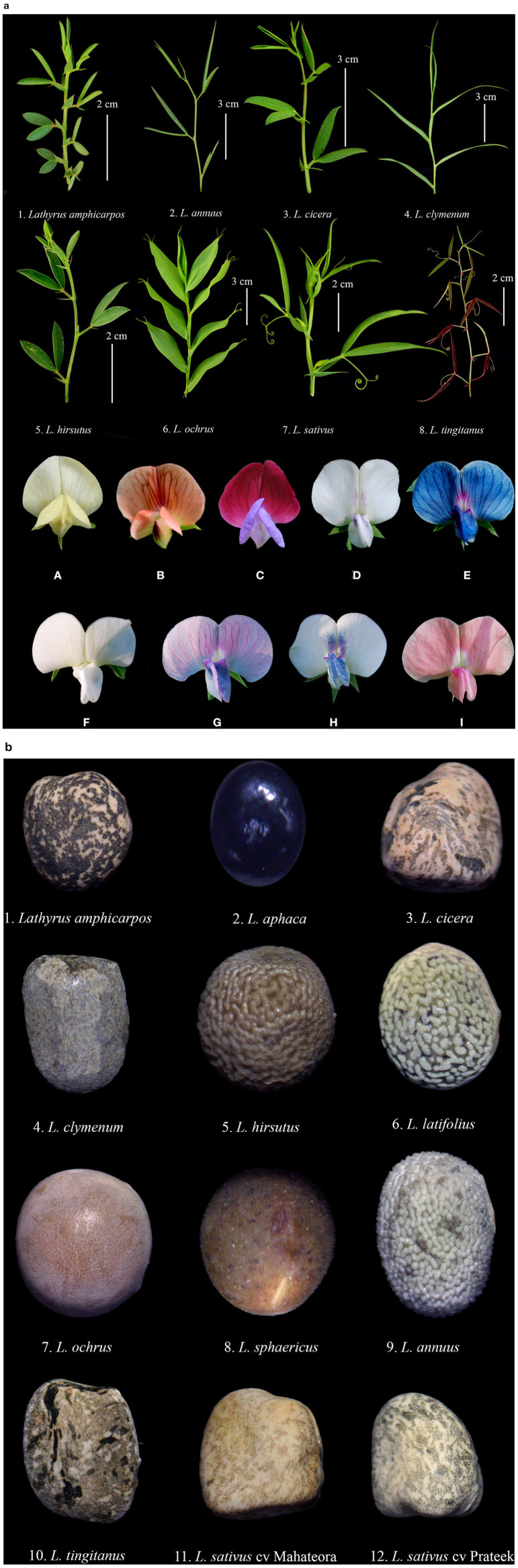
**(a)** Leaf variation in different species of *Lathyrus* (top) and flower color variation (bottom) - (A) *L. aphaca*; (B) *L. cicera*; (C) *L. odoratus*; (D–I) *L. sativus*. **(b)** Seed color variation in different species of genus *Lathyrus*.

Grasspea is a herbaceous annual with a well-developed taproot system that is greatly branched, straggling, or ascending. Small, cylindrical, branching nodules cover the rootlets. The stems are quadrangular and extremely slender, having winged margins. Pinnately opposite leaves have two or three pairs of lanceolate leaflets that terminate in a simple or branching tendril. The leaflets are sessile, entire, and cuneate at the base and acuminate at the top. The stipules are triangular to oval in shape with basal appendage. The flowers are axillary, solitary with varied colors *viz*. blue, violet blue, pink, dark pink, light yellow, white or white with purple stripes. The blue flower is the most common, and the variation in pigmentation is due to four genes (9). The peduncle is relatively long (3–5 cm) with 2-minute bracts. It is primarily a self-pollinated species, but has a high rate of out-crossing, ranging from 9.8 to 27.8%. Insects like honey bees are the main pollinators and also twisted keels with a slight opening in flowers aid in cross pollination (23, 24). Standard petals are erect and clawed. Wing petals are ovate, clawed and obtuse at the top. The keel is somewhat twisted, boat-shaped, completely split dorsally and ventrally near the base, which helps in insect pollination. The colors of the keel have a lighter shade compared to the wings with different color tinges. The stamens are diadelphous (9 + 1) and filiform, having vexillary stamens. The anthers are bright yellow in color and ellipsoid in shape. The stigmas are upturned and enlarged at the tip. The stigma is spatulate, glandular-papillate and terminal. Ovaries are sessile with 5–8 ovules. The pods are oblong, flat, and slightly bulging above the seeds, with a length of 2.5–4.5 cm, with a width of 0.6–1.0 cm, and slightly curled tips (25). The two-winged, short-beaked dorsal regions of the pod contain 3–5 small seeds. The seeds are angled, wedge-shaped, and come in various colors, including white, brownish-gray, yellow, and are spotted or mottled (26). The hilum is elliptic with yellow to pinkish yellow cotyledons. The seeds germinate hypogeally with purplish-green epicotyls. The morphological variation in leaf, flower and seeds of some species of *Lathyrus* are depicted in Figures 3a,b. As described by Hanbury et al. (27); and Jackson and Yunus (19), grasspea accessions are broadly grouped into two groups viz. (1) Blue-flowered accessions with smaller brown mixed seeds from South West and South Asia and (2) White and mixed-colored accessions from the Mediterranean region. Generally, larger white seeded genotypes yield higher than the accessions from the Indian subcontinent, including those from areas lying in between the Canary Islands to the west of the republics of the former Soviet Union. Small-seeded grasspea accessions are associated with hard seed coats and are considered more primitive like chickpea and lentil of old world.

## *Lathyrus* Genetic Resources: Diversity for Sustainability

The Himalayan region of India has a very rich genetic diversity of genus *Lathyrus* with nine different species viz. *L. aphaca* L., *L. pratensis* L., *L. sphaericus* Retz., *L. inconspicuus* L., *L. odoratus* L., *L. altaicus* Ledeb., *L. luteus* Baker., L. *imphalensis* and *L. sativus* (28–34). In the recent report published by Botanical Survey of India, there are nine taxa (eight species and one subspecies) present in India. These are having their distribution accordingly which include *L. aphaca* present throughout the country; *L. hirsutus* L. and *L. cicera* in Jammu and Kashmir; *L. laevigatus* (Waldst.& Kit.) Gren., *L. pratensis, L. humilis* (Ser.) Spreng. and *L. erectus* Lag. in Himachal Pradesh, Jammu and Kashmir, Uttarakhand; *L. odoratus* in Bihar, Gujarat, Jharkhand, Madhya Pradesh, Maharashtra, Punjab, Tamil Nadu, Uttarkhand and Uttar Pradesh; *L. sphaericus* Retz. in almost throughout India except South and North East India (35). *Lathyrus* germplasm including cultivated grasspea germplasm collections are maintained *ex-situ* at many places in the world. The major genebank collections in the world are given in the Table 1. Similarly, 73 taxa of *Lathyrus* have been described in the Flora of Turkey, out of which 22 taxa are endemic (38). The global conservation strategy of grasspea highlights the urgency of upgrading documentation systems, safe multiplication, duplication and adopting international standards for managing existing collections as a means toward a rational and effective conservation system. As a part of global backup or safety duplication, a total of 4,510 accessions of different origin with 45 other species in genus *Lathyrus* from 18 depositors are conserved in the Svalbard Global Seed Vault (39). The establishment of the “*Lathyrus* Genetic Resources Network” (40) propelled foundation for the coordinated international efforts for conservation, collection and other pre-breeding works on the grasspea in the last few decades. South Asia including India is one of the major focussed areas of grasspea cultivation. The geo-referenced map of India highlights the grasspea collecting sites indicating adaptation of crops to eastern part of India which is the most populated region of country (Figure 4). If any intervention for grasspea adaptation will be supported by all stakeholders such as farmers, scientists and policy makers in systematic and focussed approach, this crop may alleviate protein malnutrition and food insecurity of the populated region of India and South Asia.

**Table 1:** The *Lathyrus* holdings in major global genebanks.

**S.N**.	**Major genebanks**	**Total *Lathyrus* accessions with three major species conserved in different global genebanks**
1	Conservatoire botanique national Midi-Pyrénées (CBNPMP), France^*^	4,477
2	International Center for Agricultural Research in Dry Areas, Lebanon (ICARDA)^**^	4,417 (*L. sativus*−2,577, *L. aphaca*−339, *L. cicera*−216)
3	Indian Council of Agricultural Research—National Bureau of Plant Genetic Resources (ICAR-NBPGR), New Delhi, India^##^	2,622
4	Bangladesh Agricultural Research Institute (Plant Genetic Resource Centre (BARI-PGRC), Bangladesh)^#^	2,422 (*L. sativus)*
5	Instituto Nacional de Investigación Agraria (INIA), Chile ^*^	1,824
6	Australian Grains Genebank, Australia^**^	1,477 (*L. sativus-* 896, *L. cicera*−201, *L. ochrus*−122)
7	Millennium Seed Bank (MSB), Kew, England^**^	1,439 (*L. aphaca*- 226, *L. sativus-* 156, *L. hierosolymitanus*−97)
8	Ustymivka Experimental Station of Plant Production, Ukraine^**^	1,215 (*L. sativus*−782, *L. cicera*−73, *L. hirsutus*−70)
9	N.I. Vavilov All-Russian Scientific Research Institute of Plant Industry, Saint Petersburg, Russia^**^	1,207 (*L. sativus*−824, *L. cicera*−86, *L. hirsutus –* 45)
10	United States Department of Agriculture (USDA) National Plant Germplasm System^**^	871 (*L. sativus*−294, *Lathyrus. sp*.,−125, *L. odoratus-* 52)

* *Patto and Rubiales (36)*.

** *https://www.genesys-pgr.org/a/overview/v2Vd8B228KX (accessed December 2021)*.

# *Mathur et al. (37)*.

## *http://genebank.nbpgr.ernet.in/SeedBank/CropSpecieswithICECWise.aspx?CropCode=1641 (accessed January 7, 2022)*.

**Figure 4:**
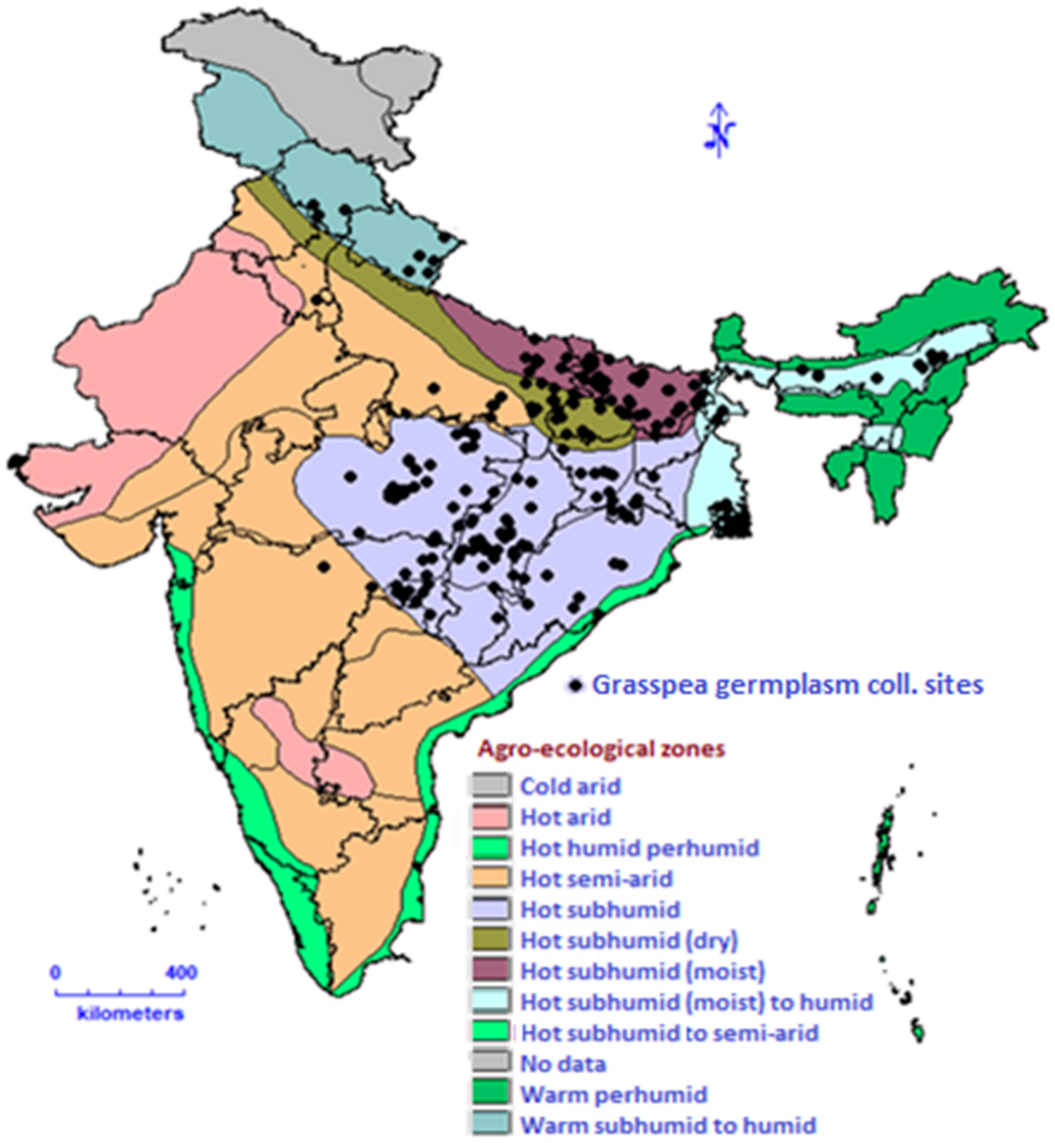
Grasspea germplasm collecting sites in India.

## Nutritional Composition of Grasspea

Grasspea seeds contain about 8.6–34.6%, protein content which is higher than chickpea (18%), field pea (21%), French bean (20%) (41). Grasspea seeds consist of around 60% globulins and 30% albumins in the total seed proteins with 90% digestibility at a 10% level of protein intake (42–46). Analysis of seeds for carbohydrates and crude fiber has shown that these vary between 48–52.3% and 1.1–1.7%, respectively (9, 27, 44). The total amino acids and fatty acids are estimated at 19.69–23.48 g/100 g and 58–80% in the same order in grasspea seeds that are in desirable proportion for animal and human consumption (47–49). Like other legumes, grasspea seeds are rich in lysine (18.4–20.4 mg/kg) but low in sulfur-containing amino acids that range 3.8–4.3 mg/kg in cysteine and 2.5–2.8 mg/kg in methionine (50, 51). Interestingly, one mutant was identified through the research showed 63% more methionine than its parent genotype. However, threonine content in seeds ranged from 10.2 to 11.5 mg/kg (47). The contribution of the total lipids, ascorbic acid and glutathione amount to 1.67 g, 13.50 mg and 15.90 mg/100 g grasspea seeds in the same order. The legume also has higher glutathione and ascorbic acid levels, which contributes to its enhanced antioxidant activity (44, 48, 49). The grasspea seeds contain 4.60 mg thiamine (B1), 2.30 mg riboflavin (B2), 16.40 mg niacin (B3), 18.40 mg pantothenic acid (B5), 5.80 mg pyridoxine (B6) and 5.40 mg/kg folic acid (B9), making them a strong source of vitamin B complex. Furthermore, ascorbic acid (42.5 mg/kg), retinol (34.9 g/kg), and carotene (323.3 g/kg) are all abundant in grasspea seeds (52). The collective nutritional profile of grasspea is given in the Table 2.

**Table 2:** Nutritional profile of grasspea (*L. sativus* L.).

**Nutritional contents**	**Range**	**Reference (s)**
Protein	8.60–34.60%	(42–46)
Globulins	>60% of the total proteins	(9, 27, 44)
Albumins	>30% of the total proteins	
Carbohydrate	48.0–52.3%	(44, 47)
Crude fiber	1.1–1.7%	
Homoarginine	6.26–20.97 g/kg	(49, 53–55)
Fatty acids (polyunsaturated)	127.39–179.39 mg/100 g	(47, 56, 57)
Amino acid	19.69–23.48 g/100 g	(44, 48, 52)
Total lipids	1.67 ± 0.18 g/100 g	
Glutathione	15.90 ± 0.10 mg/100 g	
Asparagine	0.59–5.22 mg/g seeds	(49, 58)
Retinol	34.9 μg/kg	
Carotene	323.3 μg/kg	
Thiamine (B1)	4.60 mg/kg	
Riboflavin (B2)	2.30 mg/kg	
Niacin (B3)	16.40 mg/kg	
Pantothenic acid (B5)	18.40 mg/kg	
Pyridoxine (B6)	5.80 mg/kg	
Folic acid (B9)	5.40 mg/kg,	(44, 48, 52)
Ascorbic acid	13.50–42.5 mg/kg	
Acid detergent fiber	4.3–7.3%	(47, 50, 51)
Calcium	0.07–0.12 mg/kg	
Phosphorus	0.37–0.49 mg/kg	
Lysine	18.4–20.4 mg/kg	
Threonine	10.2–11.5 mg/kg	
Methionine	2.5–2.8 mg/kg	
Cysteine	3.8–4.3 mg/kg	
Iron	6.9–8.74 mg/100 g	(46, 58–60)
Zinc	2.46–36.7 mg/100 g	
Potassium	644 mg/100 g	
Magnesium	92 mg/100 g	
Vitamin-E	40 IU /kg	(56)

Utilization of grasspea as food and feed or fodder is limited due to the presence of a plant neurotoxin called β-ODAP that is considered causal agent of muscle atrophy and lower limbs paralysis or neurolathyrism in humans, animals and poultry (50, 53, 61). Recent discovery of some health-promoting nutraceuticals shows some hidden potential of grasspea seeds (18). It is the only known dietary source of L-homoarginine which is useful to treat cardiovascular ailments, hypoxia -Alzheimer's disease and other memory-related disorders (54, 62–64). A wide range of L-homoarginine concentration (6.26–20.97 g/kg) (52–55) reduces excitation of neuronal receptors due to the biosynthesis of nitric oxide (65, 66) in our body. Asparagine content ranged from 0.59 to 5.22 mg/g of seeds desired for young children's healthy brain development and body functions (52). Daily dietary intake of asparagines and L-homoarginine from grasspea seeds could be beneficial to human health and need more systematic research (63). The iron and zinc content in grasspea seeds ranged 6.9–8.7 mg/100 g and 2.46–36.7 mg/100 g, respectively (58, 59). However, large genetic variability for iron and zinc concentration in *Lathyrus* genetic resources were observed at ICARDA breeding programme (46, 67) that can be used for biofortification or genetic improvement of grasspea. The comparative table on nutritive value of grasspea with other cool season legumes are given in the Table 3.

**Table 3:** Comparative table on nutritional values of grasspea (*L. sativus* L.) with other cool season legumes.

**Composition**	**Chickpea (*Cicer arietinum***	**Lentil (*Lens culinaris***	**Dry Peas (*Pisum***	**Field bean, Black**	**Grasspea**
	L.) (Whole)^*^	Medik.) (Whole, Brown)^*^	*sativum* L.)^*^	(*Phaseolus vulgaris* L.)^*^	
Protein (%)	18.77 ± 0.42	22.49 ± 0.58	20.43 ± 0.79	19.93	8.60–34.60^**^
Total poly unsaturated fatty acids	2,337 ± 78.2 mg/100 g	277 ± 9.70 mg/100 g	873 ± 41.50 mg/100 g	468 mg/100 g	127.39–179.39 mg/100 g^$^
Total Carotenoids	999 ± 240 μg/100 g	924 ± 89 μg/100 g	933 ± 94.10 μg/100 g	207 μg/100 g	323.30 μg/kg^#^
Iron	6.08 ± 0.27 mg/100 g	7.57 ± 0.67 mg/100 g	5.09 ± 0.45 mg/100 g	4.50 mg/100 g	6.90–8.74 mg/100 g^***^
Phosphorus	267 ± 21.9 mg/100 g	274 ± 27.40 mg/100 g	334 ± 18.30 mg/100 g	457 mg/100 g	0.37–0.49 mg/kg
Potassium	935 ± 37.9 mg/100 g	756 ± 63.60 mg/100 g	922 ± 67.40 mg/100 g	1,272 mg/100 g	644 mg/100 g^***^
Calcium	150 ± 18.3 mg/100 g	76.13 ± 9.23 mg/100 g	75.11 ± 13.93 mg/100 g	78.16 mg/100 g	0.07–0.12 mg/kg
Magnesium	160 ± 17.5 mg/100 g	101 ± 13.90 mg/100 g	123 ± 8.10 mg/100 g	197 mg/100 g	92 mg/100 g^***^
Vitamin B1	0.37 ± 0.04 mg/100 g	0.40 ± 0.07 mg/100 g	0.56 ± 0.05 mg/100 g	0.35 mg/100 g	0.46 mg/100 g^#^
Vitamin B2	0.24 ± 0.01 mg/100 g	0.22 ± 0.03 mg/100 g	0.16 ± 0.01 mg/100 g	0.07 mg/100 g	0.23 mg/100 g^#^
Vitamin B3	2.10 ± 0.06 mg/100 g	2.54 ± 0.12 mg/100 g	2.69 ± 0.15 mg/100 g	1.88 mg/100 g	1.24–2.03 mg/100 g^#^
Vitamin E	1.72 ± 0.07 mg/100 g	0.19 ± 0.02 mg/100 g	0.32 ± 0.02 mg/100 g	0.51 mg/100 g	40 IU /kg^##^
Cysteine	1.27 ± 0.09 g/100 g	1.18 ± 0.04 g/100 g	0.82 ± 0.15 g/100 g	0.59 g/100 g	3.8–4.3 mg/kg^###^
Threonine	3.55 ± 0.31 g/100 g	3.35 ± 0.05 g/100 g	3.65 ± 0.15 g/100 g	4.12 g/100 g	10.2–11.5 mg/kg^###^
Methionine	1.16 ± 0.16 g/100 g	0.84 ± 0.03 g/100 g	0.68 ± 0.19 g/100 g	1.36 g/100 g	2.5–2.8 mg/kg^###^

# *Grela et al. (58) and Arslan et al. (52)*.

## *Grela and Gunter (56)*.

### *Rotter et al. (47), Hanbury et al. (50), and Lambein and Kuo (51)*.

* *Indian Food Composition Table-2017 (41)*

** *Barpete et al. (42), Tamburino et al. (44), Girma and Korbu (43), Kumari and Kumar Jha (45), and Sengupta et al. (46)*.

*** *Sandberg (60), Urga et al. (59), Grela et al. (58), and Sengupta et al. (46)*.

$ *Arslan (57)*.

## Grasspea and the Case History of Lathyrism

Grasspea is a source of debate among agricultural scientists, nutritionists, and farmers for decades due to notoriety for being neurotoxic. Cantani of Naples coined the name “lathyrism” in 1873; however, the history of lathyrism finds its reference way back to ancient times (68). Lathyrism is a crippling disorder and it is more exacerbated when grasspea is the primary component of the human diet accounting for at least 30% of the caloric intake for about 3–4 months as a sole diet (40). Overconsumption of the seed has been linked to neurolathyrism, a neurodegenerative spastic paraparesis disorder due to neuroexcitatory β-ODAP. Zinc deficiency in the soil was found to increase the amount of β-ODAP in the seeds (69). Variable increase in the cases of neurolathyrism was observed among the people of Bangladesh and Ethiopia (70, 71). It is reported that the young human males, cattle and poultry are more affected by the disease (72–74). However, the studies on biosynthesis pathway of β-ODAP have found that it is co-regulated with serine and cysteine of the nitrogen and sulfur metabolism, respectively, that is inversely proportional to the β-ODAP accumulation and key enzyme β-cyanoalanine synthase (75). A novel cysteine synthase gene (LsCSase) has been discovered in grasspea. Under zinc-iron stress and polyethylene glycol-induced osmotic stress, this gene was up-regulated in young seedling tissues and seeds, with an elevated expression level (76). Understanding of the fundamental steps in the regulation and the biosynthesis of β-ODAP are significant in breeding new grasspea cultivars.

## Abandoned, Neglected, and Orphan Legume With Multiple Uses

Grasspea is a promising alternative for sustainable food production because of its inherent qualities, such as minimal water requirements, drought tolerance and disease resistance. Furthermore, it is a highly profitable crop for many developing countries like Bangladesh, Ethiopia, India, Nepal, and Pakistan (77, 78). There are reports claiming that grasspea and several other legumes were used as offerings to kings and in various religious and funeral ceremonies of mummies in the ancient Egypt, in contrast to the modern-day bad reputation of this crop, which makes it as the survival and subsistence food for the poorest of society (18). It is one of the most affordable and the largest source of protein next to soybean. It is a hardy crop, tolerant to both drought and flooding. It fixes 60–124 kg/ha nitrogen under dry conditions (67, 79) and contributes positively to the nitrogen requirements of its subsequent crops. Grasspea is an abandoned, neglected and underutilized crop that can be explored to isolate a number of compounds and metabolites contributing to human health. It has high folic acid that plays a vital role in erythropoiesis (the production of red blood cells) along with nucleic acid and protein synthesis. Therefore, it is essential in preventing congenital disabilities (44). The water-soluble inositol phosphoglycan (IPG) molecules from seeds of grasspea are being used in some traditional medicines to treat diabetic symptoms (80).

A Chinese group has also patented the metabolite β-ODAP from its seeds are used as a hemostatic agent following surgery (81). β-ODAP is also present in the roots of Chinese ginseng (*Panax ginseng* C.A.Mey.), which is believed to promote lifespan and is commercialized as “Dencichine” in the markets and are used in the treatment of hemorrhage and thrombopoiesis (82, 83). Some Chinese toothpaste brands also use its herbal extracts to avoid bleeding gums (18, 84). β-ODAP metabolite has also been shown to have the property of healing wounds naturally (84). Thereby, grasspea seeds have demonstrated range of therapeutic properties, indicating that they may be used as a potential medicinal or pharmaceutical crop plant of the future. The radical scavenging activity is explained by the presence of phenol phytochemicals they contain in their roots. It can be recommended for cultivation on unproductive marginal lands adjacent or close to hill slopes and during droughts to decrease soil erosion (44). Plant antioxidant mechanisms that accumulate ascorbic acid (AC), oxidized forms of AC-dehydroascorbic acid (DAA) and diketogulonic acid (DKGA) in *L. maritimus* (L.) Fr. contribute to antioxidant activity which helps in adapting to the changing environment (85). A new protein PGIP-Polygalacturonase-Inhibiting Protein from its seeds play an significant role in plant protection against fungal infections by endo polygalacturonases (EPGs), the first enzymes released by phytopathogenic fungi during plant infection (86). It can be used in crop improvement as the effective donor source of resistance to *Ascochyta* blight compared to other field pea cultivars (87, 88). The silver nanoparticles biosynthesized from grasspea species and *Stachys lavandulifolia* Vahl. can also be used as an antifungal agent against *Dothiorella sarmentorum* (89).

**Figure 5:**
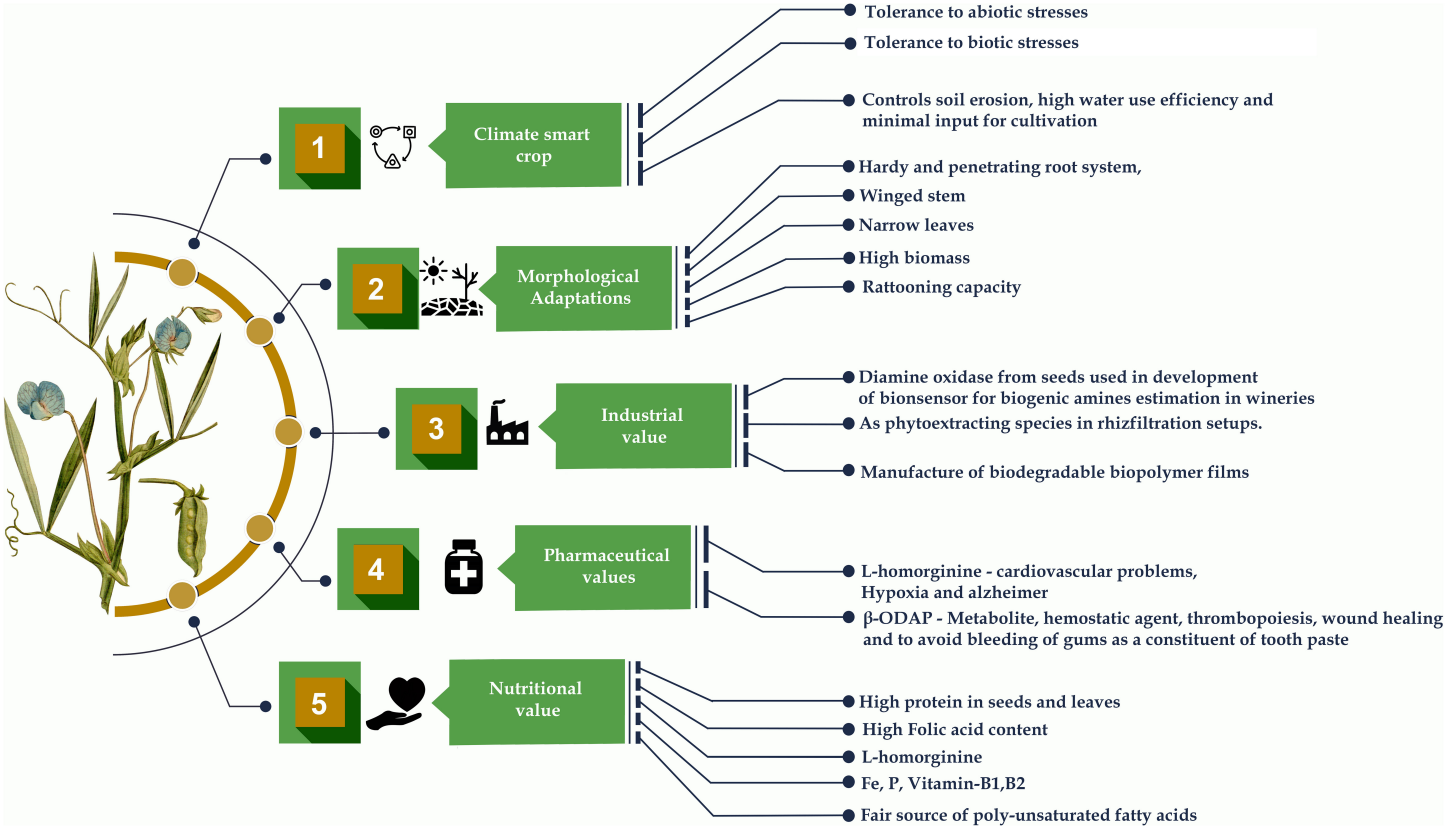
Grasspea as a multifaceted legume.

Metalloproteases isolated from the dry grasspea seeds are useful in the biotechnology, food, medical and pharmaceutical industries (90). The different proportions of grasspea protein isolates and glycerol were used to combine *Lepidium perfoliatum* L. seed gum to form composite biopolymer films that are biodegradable in nature (91). An effective biocontrol strain of plant growth promoting rhizobacteria obtained from the rhizosphere of grasspea was used as an alternative to conventional fertilizer that could contribute to crop disease reduction and significantly increase crop growth and yield (92). The ability of this crop to withstand nutrient shortage and retain vast amounts of lead in the root tissues has made it a tough species. As a result, it could be incorporated into the phytoremediation and rhizofiltration systems as an effective lead phytoextracting species (93). The characterization, evaluation and utilization of diamine oxidase (DAO) from *L. sativus* species used as biocatalytic component of a novel DAO-based amperometric electrochemical biosensor to determine biogenic amines (BA) index in the wine and beer samples. It can produce a credible assessment of the overall BA's content in production plants or wineries. Food quality commissions around the world are increasingly requesting this metric (94). Several different attractive ornamental species of genus *Lathyrus* like *L. odoratus, L. vernus* (L.) Bernh and *L. latifolius* have potential for increasing diversity in grasspea by interspecific hybridization. In the United Kingdom, a seed bank has been established to preserve the variety of ornamental plants and make the materials freely available to the academic researchers and farmers worldwide (95). Therefore, holistic research on this crop could explore the other possible and sustainable uses of the crop (Figure 5).

The seeds are generally used as a pulse, *dahl* and flour, which is used in preparing different types of savories, sweets and snacks. Immature pods and young leaves of a plant are used as a leafy vegetable in India and Bangladesh. Tender leaves and branches of the grasspea are popularly sold by the farmers in Indian and Bangladeshi markets. Apart from its use as a food, it is also used as fodder to cattle during the early vegetative stage to maturity and feed. Split-grains and flour of grasspea are also utilized as feed for lactating animals or bullocks in periods of heavy field work (15). In sustainable agriculture system, this multifaceted species can serve as an insurance crop to the marginal land farmers in drought hit areas with minimum inputs and high output. High protein contents, tolerance against the number of biotic and abiotic stresses and the ratooning (multiple cuts for its foliage) capacity of this crop makes it a good choice for cultivation in the arid regions and semi-arid regions with high scarcity of water.

## Breeding Efforts for Nutritional Gain in Grasspea

Grasspea is recognized as a versatile crop and one of the climate-smart choices for the future and it has attained the status of the multipurpose legume. With the help of conventional breeding and selection strategy from available germplasm, ICARDA and National Agricultural Research Systems (NARS) have developed and released more than 25 improved cultivars of grasspea that can be cultivated in diverse agroecology throughout the world (Table 4). Grasspea breeding programs are focussed their efforts toward the improved early maturity, high biomass, plant types, and resistance to both biotic and abiotic stresses along with low β-ODAP cultivars (46, 107, 108). This will incorporate tolerance against major pests, pathogen and induce improved nutritional quality (protein, micronutrients, methionine and homoarginine) using traditional and improved breeding protocols (25).

**Table 4:** Low-β-ODAP grasspea cultivars released in Australia, South Asia, Central Asian, Mediterranean, East African and Latin American countries.

**Breeding method**	**Variety name**	**Pedigree/selection method**	**Country**	**References**
Intraspecific hybridization	Ceora	K33 × 8604	Australia	(67, 96)
	Bari Khesari 1	P-24 × Local cultivar	Bangladesh	(67, 97)
	Bari Khesari 2	P-24 × Local cultivar	Bangladesh	(67, 97)
	Wasie (ILAT-LS-LS-B2)	SC5 × PGRC 46071	Ethiopia	(98)
	Prateek	LS 82046 × A 60	India	(99)
	Mahateora	Ratan × JRL 2	India	(99)
	Studenica	Polish cultivar × local Serbian landrace (Pedigree method)	Serbia	(67, 100)
	Stinica	Polish cultivar × local Serbian landrace (Pedigree method)	Serbia	(67, 100)
Mutation breeding	Bina Khesari 1	Mutation	Bangladesh	http://dhcrop.bsmrau.net/binakhesari-1/
Biotechnological approaches	Ratan	Somaclone of cv. Pusa-24	India	(99)
Direct introduction	CLIMA 2 pink	Introduction	Nepal	https://www.scribd.com/document/18528265/Status-of-Grasspea-in-Nepal
	Bari Khesari 2	Introduction	Bangladesh	https://nfsm.gov.in/areacoveragecropsdashboard.aspx
Direct selection from germplasm	Chalus	Selection from IFLA 1279	Australia	(67, 101)
	Bari Khesari 3	Selection from Sel.190	Bangladesh	http://dhcrop.bsmrau.net/bari-khesari-3/
	Bari Khesari 4	Selection from Sel.1337	Bangladesh	http://dhcrop.bsmrau.net/bari-khesari-4/
	Strandja	Local selection (VIL)	Bulgaria	(67)
	LS 8246	Selection from Pusa-24	Canada	(67, 102)
	Luanco-INIA	Selection from LS 0027	Chile	(67, 103)
	Quila-blanco	Selection from germplasm	Chile	(67, 104)
	Ali Bar	Selection from IFLLS- 554	Kazakhstan	(105)
	Pusa-24	Selection from germplasm	India	(99)
	Nirmal	Selection from germplasm	India	(99)
	Bidhan Khesari-1	Selection from LAT-15-6 (BK-14-1)	India	(99)
	19A	Selection from germplasm	Nepal	https://www.scribd.com/document/18528265/Status-of-Grasspea-in-Nepal
	19B	Selection from germplasm	Nepal	https://www.scribd.com/document/18528265/Status-of-Grasspea-in-Nepal
	Derek	Selection from Der	Poland	(67, 106)
	Krab	Selection from Kra	Poland	(67, 106)
	Gurbuz-1	Selection from IFLLS 554	Turkey	(98)

Characterization and breeding efforts were attempted in grasspea for agro-morphological traits and β-ODAP content (48, 109). Cross compatibility studies were carried out between common peas, *Pisum sativum* and *L. sativus*. It was observed that there was successful isolation, culture and fusion of viable protoplasts from these crop plants. This will allow for the development of genetic novelties with intriguing agronomic properties such as stress tolerance and rusticity from grasspea and grain quality from peas (110). The use of genetically distant grasspea accessions could give possible superior recombination with low β-ODAP content compared to carrying out crosses among or between the genetically closer species (111).

Mutation studies on grasspea with gamma-rays are encouraging and have shown induced salinity (NaCl) tolerance in M2 progeny mutants of grasspea (112). Different kinds of auxins (IBA; IAA; NAA) in tissue culture experiments are carried out to find the factors affecting healthy rooting, acclimatization and the effects of different concentration of sugar on root morphology phenology and developmental attributes of grasspea plants (25, 113, 114).

Grasspea has natural source of resistance to many pulse diseases (36). *Ascochyta* blight can be considered one of the most important diseases in legumes (88). *Ascochyt*a blight resistance was observed in various species of genus *Lathyrus* namely *L. cicera, L. clymenum L., L. ochrus* (L.) DC. and *L. sativus* as in comparison with the field peas (87, 115). The gene expression for creating resistance against *Ascochyta lathyri* in grasspea has also been demonstrated (88).

Limited genetic and genomic research by the public and private sector for the genus *Lathyrus* has resulted in meagre and stagnant data on the desirable aspects of grasspea. In future improvement programmes, the use of molecular markers for the genetic diversity studies and their utilization in marker-trait association for plant phenology and yield-related traits are expected to play a crucial role in understanding the association of novel alleles in trait expression (116, 117). The development and use of simple sequence repeats (SSRs) (45, 118–120), EST-SSR (111), Restriction Fragment Length Polymorphism (RFLP), and Random Amplified Polymorphic DNA (RAPD) (10, 121, 122) markers as a conventional molecular tool and recent development of SSR markers by *In silico* mining of nucleotide sequences (117) has enhanced our understanding in genetic linkage mapping, QTL mapping, association mapping, DNA fingerprinting and genetic diversity studies. The above mentioned research on the topics related to grasspea breeding has given a promising way of exploring the genetic potential of this species. In addition to this information, phylogenetic relationship between different species of the genus *Lathyrus* using chloroplast DNA trnH-psbA -intergenic spacer (123), nuclear ribosomal DNAITS2-nrDNA - Internal Transcribed Spacer 2 (124) and an Inter-Simple Sequence Repeats (ISSR) technique has been carried out (125) to know the better understanding of the existence of genetic diversity among the accessions in their experiments.

A draft genome sequence of grasspea ranged between 6.75 and 7.63 Gbp (126), 7.82 and 8.90 Gbp (127), 6.85 Gbp (128), and 6.52 Gbp (129). This data will help to identify the genes responsible for the gene regulation of biosynthesis pathway of β-ODAP and identify the alleles for different traits that will be helpful in the agronomic and nutritional improvement. It is also expected to allow comparative genomic analyses between different legumes, which will aid in the development of genetic and physical maps which can be used for the development of marker-assisted and genomic selection strategies through genome editing and tilling platforms (23).

## Current Scenario of Grasspea Cultivation and Lathyrism in India

Grasspea has the immense potential to grow as a rice-fallow pulse crop in eastern India. A study has showed that out of 11.6 million hectares of fallow land in India, ~0.5 million hectares could be easily brought under grasspea cultivation to improve land productivity and raise revenue for farmers as a second crop (130). The Indian farmers are discouraged to grow grasspea on large scale for commercial purpose, except for family consumption and livestock feed. Commercial production of grasspea is on ban in some Indian states under the Prevention of Food Adulteration Act 1961 (131). This has ended up in reduction of its farming areas from 1.3 million hectares to <850,000 ha in a decade (67). Contrary, the researchers are becoming more interested in grasspea due to its multifaceted importance. Therefore, they are interested to breed zero or low β-ODAP cultivars. It is expected that an interaction among government, breeders, farmers, pharmaceutical professionals will increase awareness about the genus *Lathyrus* and help in developing techniques to detoxify β-ODAP. This will further increase their importance as a new putative functional food (pharmaceutically valuable crop), forage and crop of industry, along with other pulse crops. Hence, abandoning or neglecting this crop may not be a wise decision. Efforts toward developing and popularization of low or zero β-ODAP cultivars would need some detoxification methods to enhance the use of grasspea in the common households. Some of the popular grasspea detoxification methods are described and listed in Table 5.

**Table 5:** Traditional and acquired knowledge based β-ODAP seed detoxifying methods of grasspea.

**Detoxification techniques**	**Methodology**	**References**
Roasting	Seeds are roasted at 180°C for 45 min.	(132)
Roasting after soaking seeds in water	Overnight soaking of seeds then roasting as described in procedure no. 1.	
Boiling in freshwater	Overnight soaking and then boiling next day.	(114, 132–135)
Soaking in alkaline water and boiling	Seeds are soaked for 6 h in a 1% calcium hydroxide solution (1:5 w/v), then wrapped in muslin fabric and boiled for 45 min. Then it is dried and pulverized as flour.	(132, 136)
Soaking in tamarind water and boiling	The seeds are steeped for 6 h in tamarind water (1:3 w/v). Then it is washed in fresh water and cooked for 45 min. After this, it can be dried and powdered to use as flour.	(132)
Germination	Germinated seedlings over a muslin cloth which takes 30–36 h for sprouting can be eaten as microgreens or salads.	
Autoclaving	Soaking seeds overnight followed by autoclaving/ pressure cooked at 15 psi for different time intervals, say 15, 30, and 45 min.	(114, 132, 135)
Frying	Overnight soaking and then deep frying in vegetable oil.	(132)
Fermentation with bacterial and fungal inoculum	Overnight soaking of seeds followed by boiling and then it is crushed in a mixer for 5 s before being placed on Petri dishes. Then this mixture is fermented with *Aspergillus oryzae* spores after sterilization (110°C, 30 min) for 48 h at 30°C. Then it is cooled and again inoculated with *Rhizopus microspores* var *chinensis* and allowed for further and fermentation for about 42 h at 30°C. Both the fungal and bacterial fermentation are inoculated with 108 spores per petri dish of each and ended with steam (100°C, 20 min). The resultant product is called as “tempeh”- a traditional fermented protein-rich product resembling cake slices.	(132, 137)

## Recent Trends on Lathyrism in India

Presently in India, about 3.62 lakh ha area of land is under grasspea cultivation (138). Several studies about grasspea consumption were conducted by Nagarajan and Gopalan (139) in Bilaspur, Durg, and Raipur districts of Chhattisgarh in India. Previous studies noted that β-ODAP content in most of the lines or cultivars ranged between 0.5 g and 2.5 g/100 g (78). These districts were restudied after 50 years in 2018. The new studies showed that β-ODAP content in local germplasm was significantly reduced and ranged 0.63 ± 0.14 to 0.65 ±0.14 g 100 g^−1^. No or negligible incidence of neurolathyrism which includes the occurrence of these symptoms in aged persons of 50–60 yrs were reported in these areas (140). Similar findings were reported from Bora and Malgaon villages along with Miraj country (Tehsil) of Sangli district in the Maharashtra, India (141). Chaurasia et al. (142) has reported only three cases of post stroke paralysis from Eastern Uttar Pradesh. However, grasspea consumption cannot be blamed solely for this report. These findings suggested a considerable reduction in the incidence of neurolathyrism compared to its reports in the past primarily due to consumption of low ß-ODAP cultivars that were distributed among the farmers by the state government.

## Future Perspective

**Figure 6:**
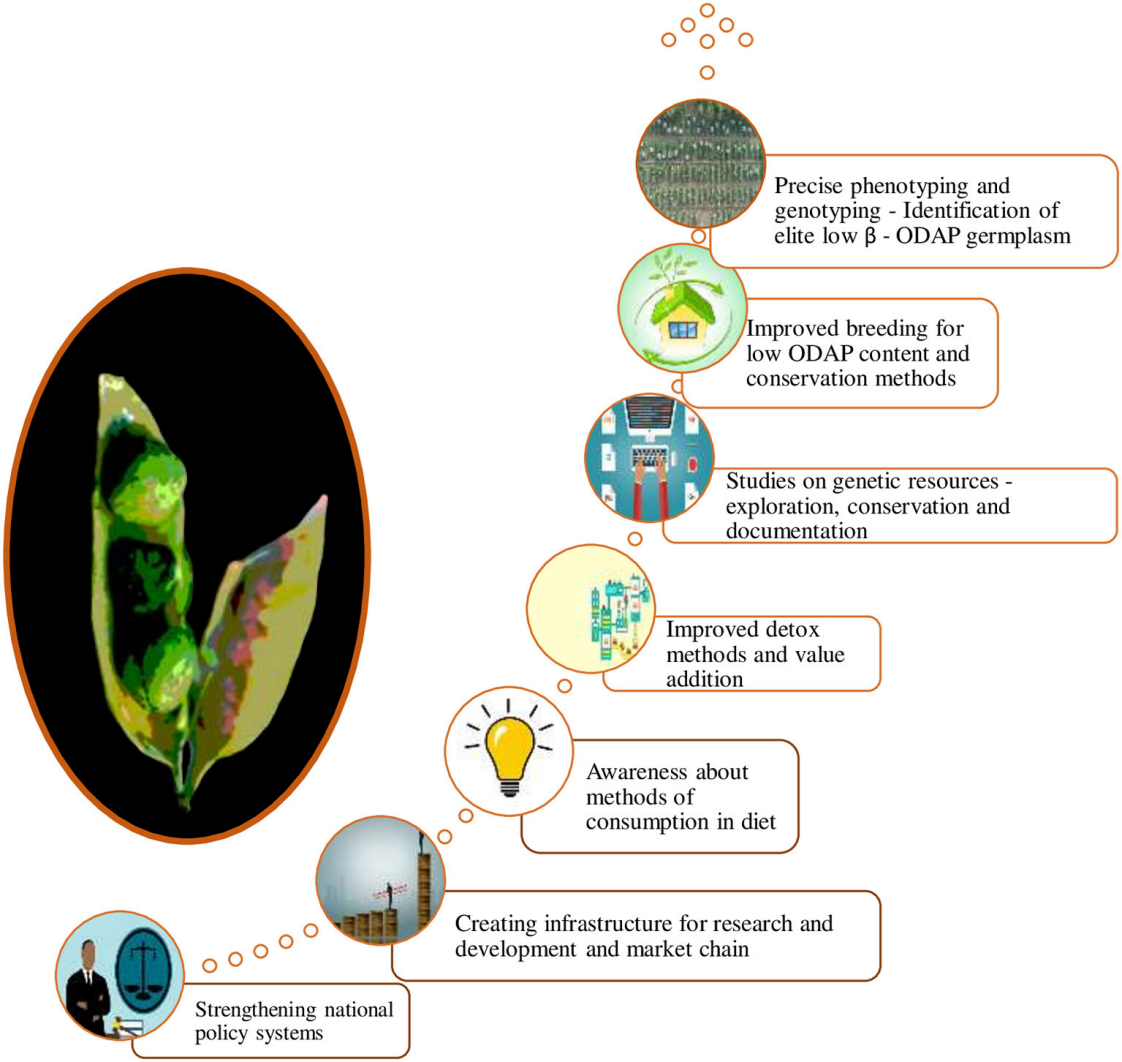
Ways to promote grasspea cultivation.

Different species in genus *Lathyrus* including cultivated grasspea have greater potential for nutritional use in the industry compared to other legumes. Therefore, Indian government and some International organizations are paying more attention and importance to the conservation and utilization of grasspea genetic resources due to their versatile uses under rapidly changing environmental conditions (78). The coordinated programme with proper methodologies for breeding new low or zero β- ODAP cultivars is desired. Similarly, development of methodologies for detoxification of β-ODAP and antinutrient contents in grasspea are the need of hour for this miraculous crop. These will boost national economy and improve standards of living of the farmers. The ideas to transform grasspea from orphan, neglected and abandoned crop to the multi-faceted mainstream crop would bring additional new sources of income to the farmers along with proper dietary consumption awareness among the people to reduce the incidences of neurolathyrism. The concepts for mainstreaming grasspea are proposed in Figure 6.

For enhancing the quality and quantity of pulses seed in the country, an Indian model of creation of seed-hubs can be replicated globally with the mandated objectives and targeted seed production of latest varieties. The farmers should be continuously provided with seeds of improved grasspea cultivars through seed distribution centers in the coming years and should be continuously educated and updated with latest information with diffusion of new dissemination technologies, encouraging them to improve seed production and multiplication technologies following available appropriate agronomic practices. It is expected that the improved government policies highlighting the paramount importance of the grasspea in the South Asian context, as a primary staple pulse for marginal farmers for their subsistence in dietary supplements will make and turn out this crop as the “Golden Pulse Crop of the Future.” Recently released draft genome of grasspea by Emmrich et al. (23) will make it easier to harness genomic information for breeding new cultivars to maximize its potential as a high protein pulse and a donor source for multiple resistance to biotic and abiotic stresses. The new and superior cultivars using this technology will facilitate farmers and poor people with small to marginal economies in more sustainable way. Inclusion of grasspea in irrigated and arid agricultural systems and its introduction to marginal lands with low input could prove it as a highly resilient, climate-smart crop in times to come.

## Author Contributions

KT, KR, and AP: conceptualization, writing, and editing of the manuscript. KR, PG, SB, AR, and NS: collection and compilation of information. KT, AP, SB, KK, and AS: conception, writing, review, and final editing. All authors contributed to the article and approved the submitted version.

## Funding

This work has funding support from the International Center for Agricultural Research in the Dry Areas (ICARDA), India Office for open access publication fees.

## Conflict of Interest

The authors declare that the research was conducted in the absence of any commercial or financial relationships that could be construed as a potential conflict of interest.

## Publisher's Note

All claims expressed in this article are solely those of the authors and do not necessarily represent those of their affiliated organizations, or those of the publisher, the editors and the reviewers. Any product that may be evaluated in this article, or claim that may be made by its manufacturer, is not guaranteed or endorsed by the publisher.

## References

[1] Arora NK. Impact of climate change on agriculture production and its sustainable solutions. Environ Sustain. (2019) 2:95–6. 10.1007/s42398-019-00078-w

[2] Grebmer K Bernstein J Alders R Dar O Kock R Rampa F. Global Hunger Index: One Decade to Zero Hunger: Linking Health and Sustainable Food Systems. Bonn: Welthungerhilfe (2020).

[3] Food Agricultural Organization of the United Nations (FAO); International Fund for Agricultural Development (IFAD); United Nations Children's Fund (UNICEF); World Food Programme (WFP); World Health Organization (WHO). The State of Food Security and Nutrition in the World 2020 and Transforming Food Systems for Affordable Healthy Diets For All. Rome: Food and Agriculture Organization of the United Nations (2020). Available online at: https://www.fao.org/3/ca9692en/online/ca9692en.html

[4] World Health Organization (2018). Available online at: https://www.who.int/news-room/fact-sheets/detail/malnutrition (accessed December 19, 2021).

[5] Global Nutrition Report. Available online at: https://globalnutritionreport.org/reports/global-nutrition-report-2018/burden-malnutrition/ (accessed December 16, 2020).

[6] Pratap A Gupta S. The Beans and the Peas: From Orphan to Mainstream Crops. Sawston: Woodhead Publishing (2020).

[7] Tripathi K Pamarthi RK Gowthami R Gore PG Gayacharan C Barpete S. Deciphering morpho-taxonomic variability in *Lathyrus* species. Ind J Plant Genet Resour. (2021) 34:279–89. 10.5958/0976-1926.2021.00027.9

[8] Loudon JC. Encyclopaedia of *Plants, new edition edited by Jane Loudon*. London: Longman, Brown, Green & Longmans (1855).

[9] Campbell CG. Grass pea, Lathyrus sativus L. Vol. 18. Rome: Bioversity International (1997).

[10] Chtourou-Ghorbel N Lauga B Combes D Marrakchi M. Comparative genetic diversity studies in the genus Lathyrus using RFLP and RAPD markers. Lathyrus Lathyrism Newsletter. (2001) 2:62−8.

[11] Plitmann U Gabay R Cohen O. Innovations in the tribe Vicieae (Fabaceae) from Israel. Israel J Plant Sci. (1995) 43:249–58. 10.1080/07929978.1995.10676609

[12] Allkin R. Names and Synonyms of Species and Subspecies in the'Vicieae': issue 3. Southhampton: Vicieae Database Project, University of Southampton, Biology Department (1986).

[13] Smartt J Kaul A Araya WA Rahman MM Kearney J. (1994) Grasspea (Lathyrus sativus L.) as a potentially safe legume food crop. In: Muehlbauer FJ, Kaiser WJ, editors. Expanding the Production and Use of Cool Season Food Legumes. Curr Plant Sci Biotechnol Agric. 19:144–55. Dordrecht: Springer. 10.1007/978-94-011-0798-3_7

[14] Rizvi AH Sarker A Dogra A. Enhancing grass pea (*Lathyrus sativus* L.) production in problematic soils of South Asia for nutritional security. Indian J Genet Plant Breed. (2016) 76:583–92. 10.5958/0975-6906.2016.00074.2

[15] Tyagi R Pandey A Agrawal A Varaprasad K Paroda R Khetarpal R. Regional Expert Consultation on Underutilized Crops for Food and Nutritional Security in Asia and the Pacific Thematic, Strategic Papers and Country Status Reports. Bangkok: Asia-Pacific Association for Agricultural Research Institutions (APAARI) (2017).

[16] Kupicha FK. The infrageneric structure of *Lathyrus*. Notes RBG Edinburgh. (1983) 41:209–44.

[17] Smartt J. Evolution of grain legumes. I. Mediterranean pulses. Exp Agric. (1984) 20:275–96. 10.1017/S001447970001796830886898

[18] Lambein F Travella S Kuo YH Montagu MV Heijde M. Grass pea (*Lathyrus sativus* L.): orphan crop, nutraceutical or just plain food. Planta. (2019) 250:821–38. 10.1007/s00425-018-03084-0 30719530

[19] Jackson MT Yunus AG. Variation in the grass pea (*Lathyrus sativus* L.) and wild species. Euphytica. (1984) 33:549–59. 10.1007/BF00021156

[20] Saraswat KS. The ancient remains of the crop plants at Atranjikhera (c. 2000-1500 B.C.). J Indian Bot Soc. (1980) 59:306–19.

[21] Renfrew JM. The archaeological evidence for the domestication of plants: methods and problems. In: Ucko PJ, Dimbleby GW, editors. The Domestication and Exploitation of Plants and Animals. London: Transaction Publishers (1969). p. 149–72.

[22] Kislev ME. Origins of the cultivation of *Lathyrus sativus* and *L. cicera* (Fabaceae). Econ Bot. (1989) 43:262–70. 10.1007/BF02859868

[23] Emmrich PM Sarkar A Njaci I Kaithakottil GG Ellis N Moore C. A draft genome of grass pea (*Lathyrus sativus*), a resilient diploid legume. bioRxiv [Preprint]. (2020). 10.1101/2020.04.24.058164

[24] Rahman MM Kumar J Rahman MA Afzal MA. Natural outcrossing in *Lathyrus sativus* L. Indian J Genet. (1995) 55:204–7.

[25] Barpete S Gupta P Singh M Kumar S. Culture selected somaclonal variants showing low-ODAP and high protein content in nineteen grass pea (*Lathyrus sativus* L.) genotypes. Plant Cell Tissue Org Cult. (2020) 142:625–34. 10.1007/s11240-020-01889-0

[26] Barpete S. Genetic associations, variability and diversity in biochemical and morphological seed characters in Indian grass pea (*Lathyrus sativus* L.) accessions. Fresen Environ Bull. (2015) 24:492−7.

[27] Hanbury CD Siddique KHM Galwey NW. Cocks PS. Genotype-environment interaction for seed yield and ODAP concentration of *Lathyrus sativus* L. and *L. cicera* L. in Mediterranean-type environments. Euphytica. (1999) 110:45–60. 10.1023/A:1003770216955

[28] Hooker JD. Flora of British India. Vol. II. London, L. Reeve & Co. (1879).

[29] Bamber CJ. Plants of the Punjab. Govt. Printing Press, Lahore. Reprinted 1976. Bishen Singh Mahendra Pal Singh, Dehradun and Periodical Experts, Delhi (1916).

[30] Sastri BN. *The Wealth of India.* A dictionary of indian raw materials and industrial products. Raw Mater. (1962) 6:36–41.16087633

[31] Babu CR. Herbaceous Flora of Dehra Dun. New Delhi-India, Publications and Information Directorate, CSIR (1977).

[32] Tiwari SDN. The Phyto-geography of Legumes of Madhya Pradesh. Dehradun: Central India (1979).

[33] Pandey RL Sharma RN Chitale MW. Status of Lathyrus genetic resources in India. Lathyrus Genet Resour Netw. (1999) 8:7

[34] Rana SK Rawat GS. Database of Himalayan plants based on published floras during a century. Data. (2017) 2:36. 10.3390/data2040036

[35] Sanjappa M. Fabaceae, in: flowering plants of india, an annotated check list, dicotyledons (edited by Mao AA, and Dash, S.S. Botan Survey of India. (2020) 1:300–446.

[36] Patto CMV Rubiales D. Lathyrus diversity: available resources with relevance to crop improvement–sativus L. *and L. cicera* as case studies. Ann Bot. (2014) 113:895–908. 10.1093/aob/mcu024 24623333 PMC3997641

[37] Mathur PN Alercia A Jain C. Lathyrus germplasm Collections Directory. Rome: Bioversity International (2005).

[38] Turkish Plants Data Service (2021). Available online at: https://www.tubives.com (accessed July 10, 2021).

[39] Svalbard Global Seed Vault. Nordgen (2021). Available online at: https://seedvault.nordgen.org/ (accessed June 20, 2021).

[40] Mathur PN Rao RV Arora RK. Lathyrus Genetic Resources Network. Rome: Bioversity International (1999).

[41] Longvah T Anantan I Bhaskarachary K Venkaiah K Longvah T. Indian Food Composition Tables. Hyderabad: National Institute of Nutrition, Indian Council of Medical Research (2017). p. 2–58.

[42] Barpete S Dhingra M Parmar D Sairkar P Sharma NC. Intraspecific genetic variation in eleven accessions of Grass Pea using seed protein profile. Sci Secure J Biotechnol. (2012) 1:21−7.

[43] Girma D Korbu L. Genetic improvement of grass pea (*Lathyrus sativus*) in Ethiopia: an unfulfilled promise. Plant Breeding. (2012) 131:231–6. 10.1111/j.1439-0523.2011.01935.x

[44] Tamburino R Guida V Pacifico S Rocco M Zarelli A Parente A. Nutritional values and radical scavenging capacities of grass pea ('*Lathyrus sativus*' L.) seeds in Valle Agricola district, Italy. Aust J Crop Sci. (2012) 6:149–56.

[45] Kumari S Jha VK Kumari D Ranjan R Nimmy MS Kumar A. Protein content of Lathyrus sativus collected from diverse locations. J Pharmacogn Phytochem. (2018) 7:1610–1.

[46] Gupta DS Barpete S Kumar J Kumar S. Breeding for Better Grain Quality in Lathyrus. In: Gupta DS, Gupta S, Kumar J, editors. Breeding for Enhanced Nutrition and Bio-Active Compounds in Food Legumes. Cham: Springer (2021). p. 131–56. 10.1007/978-3-030-59215-8_6

[47] Rotter RG Marquardt RR Campbell CG. The nutritional value of low lathyrogenic Lathyrus (*Lathyrus sativus*) for growing chicks. Br Poultry Sci. (1991) 32:1055–67. 10.1080/00071669108417429

[48] Grela ER Rybinski W Klebaniuk R Matras J. Morphological characteristics of some accessions of grass pea (*Lathyrus sativus* L.) grown in Europe and nutritional traits of their seeds. Genet Resour Crop Evol. (2010) 57:693–701. 10.1007/s10722-009-9505-4

[49] Arslan M Oten M Erkaymaz T Tongur T Kilic M Elmasulu S. β-N-oxalyl-L-2, 3-diaminopropionic acid, L-homoarginine, asparagine contents in the seeds of different genotypes *Lathyrus sativus* L. as determined by UHPLC-MS/MS. Int J Food Prop. (2017) 20(Suppl. 1):S108–18. 10.1080/10942912.2017.1289961

[50] Hanbury CD White CL Mullan BP Siddique KHM. A review of the potential of *Lathyrus sativus* L. and *L. cicera* L. grain for use as animal feed. Anim Feed Sci Technol. (2000) 87:1–27. 10.1016/S0377-8401(00)00186-3

[51] Lambein F Kuo YH. Prevention of neurolathyrism during drought. Lancet. (2004) 363:657. 10.1016/S0140-6736(04)15601-8 14987895

[52] Arslan M. Diversity for vitamin and amino acid content in grass pea (*Lathyrus sativus* L.). Legume Res Int J. (2017) 40:803–10.10.18805/LR-369. 10.18805/LR-369

[53] Piergiovanni AR Damascelli A. L-homoarginine accumulation in grass pea (*Lathyrus sativus* L.) dry seeds. A preliminary survey. Food Nutr Sci. (2011) 2:207. 10.4236/fns.2011.23028

[54] Van Wyk SG Kunert KJ Cullis CA Pillay P Makgopa ME Schlüter U. The future of cystatin engineering. Plant Science. (2016) 246:119–27. 10.1016/j.plantsci.2016.02.016 26993242

[55] Zhao R Sun HL Mei Chao. Wang XJ Yan L. The Arabidopsis Ca2+-dependent protein kinase CPK12 negatively regulates abscisic acid signaling in seed germination and post-germination growth. New Phytol. (2011) 192:61–73. 10.1111/j.1469-8137.2011.03793.x 21692804

[56] Grela ER Gunter KD. Fatty acid composition and tocopherol content of some legume seeds. Anim Feed Sci Technol. (1995) 52:325–31. 10.1016/0377-8401(94)00733-P25526984

[57] Arslan M. Fatty acid characteristics of grass pea (Lathyrus sativus) in an east mediterranean environment. Cogent Chem. (2017) 3:1296748. 10.1080/23312009.2017.1296748

[58] Grela ER Rybinski W Matras J Sobolewska S. Variability of phenotypic and morphological characteristics of some *Lathyrus sativus* L. and *Lathyrus cicera* accessions L. and nutritional traits of their seeds. Genet Resour Crop Evol. (2012) 59:1687–703. 10.1007/s10722-011-9791-5

[59] Urga K Fufa H Biratu E Husain A. Evaluation of *Lathyrus sativus* cultivated in Ethiopia for proximate composition, minerals, β-ODAP and anti-nutritional components. Afr J Food Agric Nutr Dev. (2005) 5:1–15. 10.18697/ajfand.8.1030

[60] Sandberg AS. Bioavailability of minerals in legumes. Br J Nutr. (2002) 88:281–5. 10.1079/BJN/2002718 10.1079/BJN/2002718 12498628

[61] Pascual VC Leal MJR Hurtado MC Pons RMG Gallego AJ Oliag PT. Report of the Scientific Committee of the Spanish Agency for Consumer *Affairs, Food Safety and Nutrition (AECOSAN) on the Safety of Grass Pea Flour Consumption*. Madrid: Food Safety and Nutrition (AECOSAN) (2018).

[62] Jammulamadaka N Burgula S Medisetty R Ilavazhagan G Rao S Singh SS. β-N-Oxalyl-l-α, β-diaminopropionic acid regulates mitogen-activated protein kinase signaling by down-regulation of phosphatidylethanolamine-binding protein 1. J Neurochem. (2011) 118:176–86. 10.1111/j.1471-4159.2011.07299.x 21554319

[63] Rao SC Northup BK. Grass pea (*Lathyrus sativus* L.) as a pre-plant nitrogen source for continuous conventionally tilled winter wheat. Crop Sci. (2011) 51:1325–33. 10.2135/cropsci2010.08.0455

[64] Singh SS Rao SLN. Lessons from neurolathyrism: a disease of the past & the future of *Lathyrus sativus* (Khesari dal). Indian J Med Res. (2013) 138:32. 24056554 PMC3767245

[65] Bell EA. Nonprotein amino acids of plants: significance in medicine, nutrition, and agriculture. J Agric Food Chem. (2003) 51:2854–65. 10.1021/jf020880w 12720365

[66] Dawson VL Dawson TM London ED Bredt DS Snyder SH. Nitric oxide mediates glutamate neurotoxicity in primary cortical cultures. Proc Natl Acad Sci USA. (1991) 88:6368–71. 10.1073/pnas.88.14.6368 1648740 PMC52084

[67] Kumar S Gupta P Barpete S Choukri H Maalouf F Sarkar A. “Grass pea”: the beans and the peas, from orphan to mainstream crops. In: Pratap A, Gupta S, editors. (2020). p. 273–87. Sawston: The Beans and the Peas, Woodhead Publishing.

[68] Barrow MV Simpson CF Miller EJ. Lathyrism: a review. Q Rev Biol. (1974) 49:101–28. 10.1086/408017 4601279

[69] Lambein F Haque R Khan JK Kebede N Kuo YH. From soil to brain: zinc deficiency increases the neurotoxicity of *Lathyrus sativus* and may affect the susceptibility for the motor neurone disease neurolathyrism. Toxicon. (1994) 3:461–6. 10.1016/0041-0101(94)90298-4 8053001

[70] Haimanot RT Kidane Y Wuhib E Kalissa A Alemu T Zein A. Lathyrism in rural northwestern Ethiopia: a highly prevalent neurotoxic disorder. Int J Epidemiol. (1990) 19:664–72. 10.1093/ije/19.3.664 2262262

[71] Haque A Hossain M Lambein F Bell EA. Evidence of osteolathyrism among patients suffering from neurolathyrism in Bangladesh. Nat Toxins. (1997) 5:43–6. 10.1002/(SICI)(1997)5:1<43::AID-NT7>3.0.CO;2-M 9086459

[72] Shourie KL. An outbreak of lathyrism in central India. Ind J Med Res. (1945) 33:239–47. 21017318

[73] Attal HC Kulkarni SW Choubey BS Palkar ND Deotale PG. A field study of lathyrism-HSome clinical aspects. Ind J Med Res. (1978) 67:608–15. 680903

[74] Roy DN Kisby DE Robertson RC Spencer PS. Toxicology of *Lathyrus sativus* the neurotoxin BOAA. In: Grass pea: The Threat and Promise. Proceedings of the International Network for the Improvement of Lathyrus sativus and Eradication of Lathyrism Workshop, London. (1988). p. 76–85.2547898

[75] Xu Q Liu F Chen P Jez JM Krishnan HB. β-N-Oxalyl-L-α, β-diaminopropionic acid (β-ODAP) content in Lathyrus sativus: the integration of nitrogen and sulfur metabolism through β-cyanoalanine synthase. Int J Mol Sci. (2017) 18:526. 10.3390/ijms18030526 28264526 PMC5372542

[76] Chakraborty S Mitra J Samanta MK Sikdar N Bhattacharyya J Manna A. Tissue specific expression and in-silico characterization of a putative cysteine synthase gene from *Lathyrus sativus* L. Gene Exp Patterns. (2018) 27:128–34. 10.1016/j.gep.2017.12.001 29247850

[77] Sarwar CDM Malek MA Sarker A Hassan MS. Genetic resources of grasspea (*Lathyrus sativus* L.) in Bangladesh. In: Lathyrus Genetic Resources in Asia: Proceedings of a Regional Workshop, 27-29 December. 1995. Raipur: Indira Gandhi Agricultural University; New Delhi: IPGRI Office for South Asia (1996). 13 p.

[78] Kumar S Bejiga G Ahmed S Nakkoul H Sarker A. Genetic improvement of grass pea for low neurotoxin (β-ODAP) content. Food Chem Toxicol. (2011) 49:589–600. 10.1016/j.fct.2010.06.051 20659523

[79] Schulz S Keatinge JDH Wells GJ. Productivity and residual effects of legumes in rice-based cropping systems in a warm-temperate environment: I. Legume biomass production and N fixation. Field Crops Res. (1999) 61:23–35. 10.1016/S0378-4290(98)00146-4

[80] Paneda C Villar AV Alonso A Goni FM Varela F Brodbeck U. Purification and characterization of insulin-mimetic inositol phosphoglycan-like molecules from grass pea (Lathyrus sativus) seeds. Mol Med. (2001) 7:454–60. 10.1007/BF03401850 11683370 PMC1950051

[81] Lan G Chen P Sun Q Fang S. Methods for Treating Hemorrhagic Conditions. U.S. Patent. 8,362,081. Washington, DC: Patent US. and Trademark Office (2013).

[82] Kuo YH Ikegami F Lambein F. Neuroactive and other free amino acids in seed and young plants of *Panax ginseng*. Phytochemistry. (2003) 62:1087–91. 10.1016/S0031-9422(02)00658-1 12591261

[83] Ding S Wang M Fang S Xu H Fan H Tian Y. D-dencichine regulates thrombopoiesis by promoting megakaryocyte adhesion, migration and proplatelet formation. Front Pharmacol. (2018) 9:297. 10.3389/fphar.2018.00297 29666579 PMC5891617

[84] Sharma D Singh P Singh SS. β-N-oxalyl-l-α β-Diaminopropionic acid induces wound healing by stabilizing HIF-1α and modulating associated protein expression. Phytomedicine. (2018) 44:9–19. 10.1016/j.phymed.2018.04.024 29895497

[85] Maslennikov P Golovina E Artemenko A. Ecological and geochemical conditions for the accumulation of antioxidants in the leaves of *Lathyrus maritimus* (L.) Bigel. Plants. (2020) 9:746. 10.3390/plants9060746 32545748 PMC7356220

[86] Tamburino R Chambery A Parente A Maro AD. A novel polygalacturonase-inhibiting protein (PGIP) from *Lathyrus sativus* L. seeds. Prot Peptide Lett. (2012) 19:820–5. 10.2174/092986612801619561 22762184

[87] Gurung AM Pang ECK Taylor PWJ. Examination of Pisum and Lathyrus species as sources of ascochyta blight resistance for field pea (*Pisum sativum*). Aust Plant Pathol. (2002) 31:41−5. 10.1071/AP0106928948418

[88] Almeida NF Krezdorn N Rotter B Winter P Rubiales D VazPatto MC. Lathyrus sativustranscriptome resistance response to *Ascochyta lathyri* investigated by deepSuperSAGE analysis. Front Plant Sci. (2015) 6:178. 10.3389/fpls.2015.00178 25852725 PMC4367168

[89] Azizi Z Pourseyedi S Khatami M Mohammadi H. Stachys lavandulifolia and Lathyrus sp. mediated for green synthesis of silver nanoparticles and evaluation its antifungal activity against *Dothiorella sarmentorum*. J Clust Sci. (2016) 27:1613–28. 10.1007/s10876-016-1024-9

[90] Ramakrishna V Rajasekhar S Sudarsana LR. Identification and purification of metalloprotease from dry grass pea (*Lathyrus sativus* L.) seeds. Appl Biochem Biotechnol. (2010) 160:63–71. 10.1007/s12010-009-8523-1 19156362

[91] Ebrahimi SE Koocheki A Milani E Mohebbi M. Interactions between Lepidium perfoliatum seed gum–Grass pea (Lathyrus sativus) protein isolate in composite biodegradable film. Food Hydrocoll. (2016) 54:302–14. 10.1016/j.foodhyd.2015.10.020

[92] Mussa A Million T Assefa F. Rhizospheric bacterial isolates of grass pea (*Lathyrus sativus* L.) endowed with multiple plant growth promoting traits. J Appl Microbiol. (2018) 125:1786–801. 10.1111/jam.13942 29869437

[93] Brunet J Repellin A Varrault G Terryn N Fodil YZ. Lead accumulation in the roots of grass pea (*Lathyrus sativus* L.): a novel plant for phytoremediation systems. Comptes Rendus Biol. (2008) 331:859–64. 10.1016/j.crvi.2008.07.002 18940701

[94] Di Fusco M Federico R Boffi A Macone A Favero G Mazzei F. Characterization and application of a diamine oxidase from *Lathyrus sativus* as component of an electrochemical biosensor for the determination of biogenic amines in wine and beer. Anal Bioanal Chem. (2011) 401:707–16. 10.1007/s00216-011-5131-z 21644017

[95] Parsons R Mikic A. Conservation and breeding of ornamental Lathyrus species. Ratarstvo Povrtarstvo. (2011) 48:1–6. 10.5937/ratpov1101001P24623333

[96] Siddique KHM Loss SP Herwig SP Wilson JM. Growth, yield and neurotoxin (ODAP) concentration of three Lathyrus species in Mediterranean-type environments of Western Australia. Aust J Exp Agric. (1996) 36:209–18. 10.1071/EA996020928948418

[97] Malek MA Sarwar CDM Sarker A Hassan MS. Status of grasspea research and future strategy in Bangladesh. In: Lathyrus Genetic Resources in Asia: Proceedings of a Regional Workshop, 27-29 December 1995. Raipur: Indira Gandhi Agricultural University; New Delhi: IPGRI Office for South Asia (1996). 7 p.

[98] ICARDA. ICARDA Annual Report 2006. International Center for Agricultural Research in the Dry Areas, Aleppo (2007). p. 57–8.

[99] ICAR. Project Coordinator's Report of All India Coordinated Research Project on Mungbean, Urdbean, Lentil, Lathyrus, Rajmash, and Pea. Indian Council of Agricultural Research (ICAR), New Delhi (2009). p 18.

[100] Mikic A Mihailovic V Cupina B Duric B Krstic D Vasic M. Towards the re-introduction of grass pea (Lathyrus sativus) in the West Balkan Countries: the case of Serbia and Srpska (Bosnia and Herzegovina). Food Chem Toxicol. (2011) 49:650–4. 10.1016/j.fct.2010.07.052 20696197

[101] White CL Hanbury CD Young P Phillips N Wiese SC Milton JB. The nutritional value of Lathyrus cicera and Lupinus angustifolius grain for sheep. Anim Feed Sci Technol. (2002) 99:45–64. 10.1016/S0377-8401(02)00035-4

[102] Campbell CG Briggs CJ. Registration of low neurotoxin content *Lathyrus* germplasm LS 8246. Crop Sci. (1987) 27:820–1. 10.2135/cropsci1987.0011183X002700040055x

[103] Mera M Tay J France A Montenegro A Espinoza N Gaete N. Luanco-INIA, a large-seeded cultivar of Lathyrus sativus released in Chile. Lathyrus Lathyrism Newsletter. (2003) 3:26.

[104] Campbell CG Mehra RB Agrawal SK Chen YZ Moneim AMA Khawaja HIT. Current status and future strategy in breeding grasspea (*Lathyrus sativus*). In: Expanding the Production and Use of Cool Season Food Legumes. Dordrecht: Springer (1994). p. 617–30.

[105] ICARDA. ICARDA Annual Report 2005. International Center for Agricultural Research in the Dry Areas, Aleppo (2006). p. 54–5.

[106] Milczak M Pedzinski M Mnichowska H Szwed-Urbas K Rybinski W. Creative breeding of grasspea (*Lathyrus sativus* L.) in Poland. Lathyrus Lathyrism Newsletter. (2001) 2:85−8.

[107] Kumar S Gupta P Barpete S Sarker A Amri A Mathur PN. Grass Pea. Genetic and Genomic Resources of Grain Legume Improvement. London: Elsevier (2013). p. 269–92. 10.1016/B978-0-12-397935-3.00011-6

[108] Singh M Upadhyaya HD Bisht IS. Genetic and Genomic Resources of Grain Legume Improvement. London: Elsevier Inc. (2013). 10.1016/C2012-0-00217-7

[109] Tavoletti S Iommarini L Crino P Granati E. Collection and evaluation of grasspea (*Lathyrus sativus* L.) germplasm of central Italy. Plant Breeding. (2005) 124:388–91. 10.1111/j.1439-0523.2005.01125.x

[110] Durieu P Ochatt SJ. Efficient intergeneric fusion of pea (*Pisum sativum* L.) and grass pea (*Lathyrus sativus* L.) protoplasts. J Exp Bot. (2000) 51:1237–42. 10.1093/jxb/51.348.1237 10937699

[111] Arslan M Basak M Aksu E Uzun B Yol E. Genotyping of Low β-ODAP Grass Pea (*Lathyrus sativus* L.) germplasm with EST-SSR markers. Braz Arch Biol Technol. (2020) 63:1–13.

[112] Talukdar D. Isolation and characterization of NaCl-tolerant mutations in two important legumes, *Clitoria ternatea* L. and *Lathyrus sativus* L.: Induced mutagenesis and selection by salt stress. J Med Plants Res. (2011) 5:3619–28. 10.5897/JMPR.9000836

[113] Barpete S Khawar KM Ozcan S. Differential competence for in vitro adventitous rooting of grass pea (*Lathyrus sativus* L.). Plant Cell Tissue Organ Cult. (2014) 119:39–50. 10.1007/s11240-014-0512-6

[114] Barpete S Gupta P Khawar KM Kumar S. Effect of cooking methods on protein content and neurotoxin (β-ODAP) concentration in grass pea (*Lathyrus sativus* L.) grains. CyTA J Food. (2021) 19:448–56. 10.1080/19476337.2021.1915879

[115] Patto MCV Skiba B Pang ECK Ochatt SJ Lambein F Rubiales D. *Lathyrus* improvement for resistance against biotic and abiotic stresses: from classical breeding to marker assisted selection. Euphytica. (2006) 147:133–47. 10.1007/s10681-006-3607-2

[116] Chowdhury MA Slinkard AE. Genetic diversity in grasspea (*Lathyrus sativus* L.). Genet Resour Crop Evol. (2000) 47:163–9. 10.1023/A:100876060499018274291

[117] Soren KR Konda AK Gangwar P Tiwari VA Shanmugavadivel PS Parihar AK. Development of SSR markers and association studies of markers with phenology and yield-related traits in grass pea (*Lathyrus sativus*)." *Crop Past Sci*. (2020) 71:768–75. 10.1071/CP1955728948418

[118] Lioi L Sparvoli F Sonnante G Laghetti G Lupo F Zaccardelli M. Characterization of Italian grasspea (*Lathyrus sativus* L.) germplasm using agronomic traits, biochemical and molecular markers. Genet Resour Crop Evol. (2011) 58:425–37. 10.1007/s10722-010-9589-x

[119] Yang T Jiang J Burlyaeva M Hu J Coyne CJ Kumar S. Large-scale microsatellite development in grasspea (*Lathyrus sativus* L.), an orphan legume of the arid areas. BMC Plant Biol. (2014) 14:1–12. 10.1186/1471-2229-14-65 24635905 PMC4003855

[120] Wang F Yang T Burlyaeva M Li L Jiang J Fang L. Genetic diversity of grasspea and its relative species revealed by SSR markers. PLoS ONE. (2015) 10:e0118542. 10.1371/journal.pone.0118542 25793712 PMC4368647

[121] Croft AM Pang ECK Taylor PWJ. Molecular analysis of *Lathyrus sativus* L. (grasspea) and related Lathyrus species. Euphytica. (1999) 107:167–76. 10.1023/A:100352072137524203465

[122] Barik DP Acharya L Mukherjee AK Chand PK. Analysis of genetic diversity among selected grasspea (*Lathyrus sativus* L.) genotypes using RAPD markers. Zeitschrift für Naturforschung C. (2007) 62:869–74. 10.1515/znc-2007-11-1215 18274291

[123] Maroua G Nadia Z Imen F Neila TF Sonia M. Molecular characterization of Lathyrus species using chloroplast DNA trnH-psbA. Biochem Syst Ecol. (2014) 57:439–44. 10.1016/j.bse.2014.09.002

[124] Marghali S Fadhlaoui I Gharbi M Zitouna N Trifi-Farah N. Utility of ITS2 sequence data of nuclear ribosomal DNA: molecular evolution and phylogenetic reconstruction of Lathyrus spp. Sci Hortic. (2015) 194:313–9. 10.1016/j.scienta.2015.08.030

[125] Ghorbel M Marghali S Trifi-Farah N Chtourou-Ghorbel N. Phylogeny of Mediterranean Lathyrus species using inter simple sequence repeats markers. Acta Bot Gallica. (2014) 161:91–8. 10.1080/12538078.2013.878854

[126] Ghasem K Danesh-Gilevaei M Aghaalikhani M. Karyotypic and nuclear DNA variations in *Lathyrus sativus* (Fabaceae). Caryologia. (2011) 64:42–54. 10.1080/00087114.2011.10589763

[127] Ochatt SJ Conreux C Jacas L. Flow cytometry distinction between species and between landraces within Lathyrus species and assessment of true-to-typeness of in vitro regenerants. Plant Systemat Evol. (2013) 299:75–85. 10.1007/s00606-012-0704-7

[128] Nandini AV Murray BG Obrien IEW Hammett KRW. Intra-and interspecific variation in genome size in Lathyrus (Leguminosae). Bot J Linnean Soc. (1997) 125:359–66. 10.1111/j.1095-8339.1997.tb02265.x

[129] Macas J Novak P Pellicer J Cizkova J Koblizkova A Neumann P. In depth characterization of repetitive DNA in 23 plant genomes reveals sources of genome size variation in the legume tribe Fabeae. PLoS ONE. (2015) 10:e0143424. 10.1371/journal.pone.0143424 26606051 PMC4659654

[130] International Center for Agricultural Research in Dry Areas, ICARDA (2015). Available online at: https://www.icarda.org/media/news/grasspea-back-menu-indias-agriculture (accessed January 14, 2021).

[131] Arora RK Mathur PN Riley KW Adham Y. Lathyrus genetic resources in asia. In: Proceedings of a Regional Workshop, 27-29 December 1995. Raipur: Indira Gandhi Agricultural University; New Delhi: IPGRI Office for South Asia (1996).

[132] Yerra S Swathi P Kilari EK. Detoxification of ODAP in Lathyrus sativus by various food processing techniques. Pharm Biol Eval. (2015) 2:152−9.20850494

[133] Padmajaprasad V Kaladhar M Bhat RV. Thermal isomerisation of β-N-oxalyl-L-α, β-diaminopropionic acid, the neurotoxin in *Lathyrus sativus*, during cooking. Food Chem. (1997) 59:77–80. 10.1016/S0308-8146(96)00166-5

[134] Rao SLN. Do we need more research on neurolathyrism. Lathyrus Lathyrism Newsletter (2001) 23:2.

[135] Yerra S Sankar DG. Proximate composition of the seeds of *Lathyrus sativus* from some States of India. J Global Trends Pharm Sci. (2014) 5:1817–21.

[136] Jahan K Ahmad K. Detoxification of *Lathyrus sativus*. Food Nutr Bull. (1984) 6:1–2. 10.1177/156482658400600213

[137] Kuo YH Bau HM Rozan P Chowdhury B Lambein F. Reduction efficiency of the neurotoxin β-ODAP in low-toxin varieties of *Lathyrus sativus* seeds by solid state fermentation with *Aspergillus oryzae* and Rhizopus microsporusvarchinensis. J Sci Food Agric. (2000) 80:2209–15. 10.1002/1097-0010(200012)80:15<2209::AID-JSFA773>3.0.CO;2-W

[138] Department Department of Agriculture Farmers Welfare National Food Security Mission. Lathyrus. (2021). Available online at: https://nfsmgov.inareacoveragecropsdashboard.aspx (accessed December 19, 2021).

[139] Nagarajan V Gopalan C. Variation in the neurotoxin β-(N)-oxalylamino-alanine content in Lathyrus sativus samples from Madhya Pradesh. Ind J Med Res. (1968) 56:95–9. 5666054

[140] Khandare AL Kumar RH Meshram II Arlappa N Laxmaiah A Venkaiah K. Current scenario of consumption of Lathyrus sativus and lathyrism in three districts of Chhattisgarh State, India. Toxicon. (2018) 150:228–34. 10.1016/j.toxicon.2018.06.069 29908260

[141] Khandare AL Babu JJ Ankulu M Aparna N Shirfule A Rao GS. Grass pea consumption & present scenario of neurolathyrism in Maharashtra State of India. Indian J Med Res. (2014) 140:96. 25222783 PMC4181167

[142] Chaurasia RN Pathak A Singh S Joshi D Mishra VN. Study of knowledge, attitude, and practice in participants with regular intake of Lathyrus, but no spastic paraparesis. J Neurosci Rural Pract. (2018) 9:011–3. 10.4103/jnrp.jnrp_305_17 29456338 PMC5812133

